# Patients’ and Relatives’ Experiences of Delirium in the Intensive Care Unit—A Qualitative Study

**DOI:** 10.3390/ijerph191811601

**Published:** 2022-09-15

**Authors:** Sandra Lange, Wioletta Mędrzycka-Dąbrowska, Adriano Friganović, Dorota Religa, Sabina Krupa

**Affiliations:** 1 Department of Internal and Pediatric Nursing, Medical University of Gdańsk, Dębinki 7, 80-211 Gdańsk, Poland; 2 Department of Anaesthesiology Nursing & Intensive Care, Faculty of Health Sciences, Medical University of Gdańsk, Dębinki 7, 80-211 Gdańsk, Poland; 3 Department of Anesthesiology and Intensive Medicine, University Hospital Centre Zagreb, 10000 Zagreb, Croatia; 4 Department of Nursing, University of Applied Health Sciences, Mlinarska Cesta 38, 10000 Zagreb, Croatia; 5 Department of Neurobiology, Care Sciences and Society (NVS), Karolinska Institute, 17177 Stockholm, Sweden; 6 Institute of Health Sciences, College of Medical Sciences, University of Rzeszow, Warzywna 1A, 35-310 Rzeszow, Poland

**Keywords:** delirium, patients, family relations, family-centered care

## Abstract

(1) Introduction: Delirium is a cognitive disorder that affects up to 80% of ICU patients and has many negative consequences. The occurrence of delirium in an ICU patient also negatively affects the relatives caring for these patients. The aim of this study was to explore patients’ and their families’ experiences of delirium during their ICU stay. (2) Method: The study used a qualitative design based on phenomenology as a research method. A semi-structured interview method was used to achieve the aim. The responses of patients and their families were recorded and transcribed, and the data were coded and analyzed. (3) Results: Eight interviews were conducted with past ICU patients who developed delirium during hospitalization and their family members. The mean age of the participants was 71 years. Of the eight patients, 2 (25%) were female and 6 (75%) were male. The relationships of the 8 carers with the patients were wife (in 4 cases), daughter (in 2 cases), and son (in 2 cases). The average length of time a patient stayed in the ICU was 24 days. The following themes were extracted from the interviews: education, feelings before the delirium, pain, thirst, the day after, talking to the family/patient, and return home. (4) Conclusions: Post-delirium patients and their families feel that more emphasis should be placed on information about delirium. Most patients feel embarrassed and ashamed about events during a delirium episode. Patients fear the reaction of their families when delirium occurs. Patients’ families are not concerned about their relatives returning home and believe that the home environment will allow them to forget the delirium events more quickly during hospitalization.

## 1. Introduction

Delirium is defined as an acute cognitive disturbance accompanied by fluctuations in mental status and impaired attention and consciousness [1,2]. This complication is associated with increased duration of mechanical ventilation, is an independent predictor of mortality, and increases the length of ICU stay [3,4,5]. In addition, delirium is associated with long-term cognitive impairment in survivors of critical illness [6]. It is estimated that delirium affects up to 80% of ICU patients [7]. One of the main determinants of delirium is old age [8]. The effectiveness of pharmacological interventions in the prevention and treatment of delirium remains unclear [9]. Therefore, there is a need to develop a safe and effective management strategy. The cause of delirium is multifactorial, so multicomponent non-pharmacological interventions are a promising strategy for delirium prevention [10,11]. Studies have confirmed that family interventions reduce the incidence of delirium [12,13]. Flexible visiting arrangements have been associated with a lower incidence of delirium and lower severity of anxiety symptoms among ICU patients. In addition, family involvement in the therapeutic process had positive effects on ICU patients and on the family itself [14]. Involvement of family members in the care of ICU patients increases overall family satisfaction, satisfaction with the decision-making process, and satisfaction with the quality of care [15]. Family involvement in the ICU patient care process has recently been added to the ABCDEF package. The ABCDEF package is an evidence-based guide to the organizational changes needed to optimize recovery and patient outcomes in the intensive care unit. It includes: Assess, Prevent, and Manage Pain, Both Spontaneous Awakening Trials (SAT) and Spontaneous Breathing Trials (SBT), Choice of analgesia and sedation, Delirium: Assess, Prevent, and Manage, Early mobility and Exercise, and Family engagement and empowerment [16]. Studies have shown that greater compliance with the ABCDEF package was independently associated with improved survival and more delirium and coma-free days [17]. The Patient Centered Care (PCC) and Family Centered Care (FCC) model is an idea that is becoming more common and desirable in the medical community [18,19]. It implies the implementation of patient care that considers and respects the patient’s beliefs, values, and preferences, and involves the family in the care of the relative. Families of critically ill patients are often included in the decision-making process as representatives of critically ill patients. Therefore, the PCC and FCC model of care may be particularly useful in the ICU setting [19]. There is a growing literature of studies focusing on the experiences of caregivers whose relatives have experienced delirium while in the ICU. A study by Bohart et al. found that families have little knowledge of delirium, but in the long term they request information. This gives them a feeling of relief and understanding [20]. In the Huang et al. study, families whose relatives had delirium experienced uncertainty, fear and anxiety [21]. In our study, we also included the perspective and experiences of ICU patients. Understanding the feelings and experiences of caregivers can contribute to developing nursing interventions with patient families, providing support, and improving the relationship between medical staff and patient families. This may contribute to increased family involvement in delirium prevention interventions, improving the quality of patient care, and enhancing the wellbeing of patients as well as their families. We think that the use of a qualitative research method is an appropriate method to reveal the feelings of patients and their families related to delirium, which could not be represented by conducting quantitative research.

### Aim

The aim of this study was to explore patients’ and families’ experiences of delirium during their stay in the ICU.

## 2. Methods

A qualitative approach was used in the study and was based on phenomenology. This is a research method used to study experiences from a subjective point of view [22,23]. Phenomenological research enables the description and understanding of human experience and allows researchers to obtain in-depth insights and associated meanings [23]. Phenomenology was used in this study as a research method to describe the experiences of ICU patients and their relatives related to the occurrence of delirium. Understanding the perspectives of patients and their families is key to showing that their experiences are important, real, and lived.

### 2.1. Sample and Recruitment

The study was conducted between December 2021 and January 2022. Recruitment for the study took place in one hospital with the status of a teaching hospital with the highest level of reference in south-eastern Poland, in accordance with the Regulation of the Minister of Health of 16 December 2016 on the organizational standard of healthcare in anesthesiology and intensive care. Patients were selected purposively. The study was approved by Institutional Review Boards No. 2018/04/04. Each patient received an explanation of the purpose of the study, followed by informed written consent from the patients and verbal consent from a family member. Anonymity and confidentiality were also ensured. Respondents were recruited by two nurses in consultation with doctors who confirmed the diagnosis of delirium during the patient’s hospitalization. The nurses were trained to collect the survey interview and were skilled in working with delirium detection tools, e.g., NuDESC. The nurses had a specialization in anesthetic and intensive care nursing. The unit was used to screen for delirium problems in the context of nursing care. The NuDESC scale, which was translated and adapted to Polish conditions (NuDESC PL), was used to assess delirium. According to the results of the study, the translation of the tool is correct, comprehensible to the respondents and can be used in Poland [24]. It is a five-item scale comprising five aspects assessed in relation to delirium symptoms and the development of the disorder. Delirium was diagnosed when the score was ≥2. Inclusion criteria for the study included age 18 years or older, admission to the intensive care unit, ability to communicate in Polish (including no hearing impairment), residence within 50 km of the hospital, and a positive test for delirium according to the NuDESC PL scale. Families who observed delirium in a patient during hospitalization were included in the study. We considered a family member as someone who knew the patient well, according to a short selection process that included living with the patient or having face-to-face contact at least once a week. Patients who had a diagnosis of dementia were excluded from the study. Patients and families who did not consent to participate in the study were also excluded. We planned to study 15 pairs, but not all family members agreed to participate in the study.

### 2.2. Questionnaire Development

A semi-structured interview was used as the method to achieve the objectives. Two researchers conducted the interviews using a pre-designed questionnaire. Two nurses conducted the interviews. They were trained to collect the interview in the scope of the study and were skilled in working with delirium detection tools, e.g., NuDESC, when conducting another study on the validation of this tool. The questions in the questionnaire were developed from relevant literature. The questionnaire consisted of 5 questions addressed to the patients and 6 questions addressed to the family. The questionnaire is presented in a Table 1. The development of the interview allowed the interviewers to ask additional in-depth questions. The original interview questionnaire was written in Polish, which for the purpose of this article was translated using a standard method of translation into English. In the questionnaire, the term delirium was expressed by the term emotional disorder to make it easier for respondents to understand. Descriptions of the experiences associated with an episode of delirium are often expressed metaphorically and are always associated with emotions. Patients and their families use mild, non-clinical terms to describe the disorders associated with delirium, in contrast to medical staff for who synonyms of delirium are, e.g., disorientation, disturbance of consciousness [25]. Therefore, we considered the term “emotional disorder” to be appropriate and understandable for the study participants. First of all, the purpose of the study and what it entails was explained to each participant before consenting to take part in the study. In addition, the researchers who interviewed the participants are specialists in delirium and have experience of working in an intensive care environment. When there were concerns that patients/relatives were connecting their descriptions with other events, these were detected, and participants were directed on the events taking place during the delirium episode.

### 2.3. Data Collection

We used semi-structured interviews to gather information about patients’ and families’ experiences of delirium during hospitalization. Semi-structured interviews are the most popular method to achieve the objective. They allow researchers and participants to engage in real-time dialogue [22]. Interviews were conducted by two nurses in Polish. Prior to data collection, areas were identified to be discussed with each participant, such as: ‘education’, ‘feelings before the delirium’, ‘pain’, ‘thirst’, ‘the day after’, ‘talking to the family/patient’, and ‘return home’. Sample questions were prepared for each of these areas, which encouraged participants to share their memories and experiences. The researchers discussed the areas mentioned with each participant and asked additional questions to deepen the themes described by the participants. The interviews took place after the patient was discharged from the ward. The researchers asked patients who met the inclusion criteria to contact them 1 month after discharge from hospital (to allow sufficient recovery time for the qualitative interview). For patients unable to give consent, the researcher obtained verbal consent from the patients to approach the next of kin to obtain verbal consent to contact the patient after hospital discharge. The researchers discussed the topic and purpose of the study with the participants. Each consented to the recording of the interview and the use of the data for the research paper. The study took place face-to-face. Each interview lasted 30 min. In conducting the interviews, the researchers adopted an open approach, which is key to the phenomenological approach. This means that the researcher opens to the phenomenon as it presents itself. When the research participants started to feel uncomfortable or became very emotional the interviewer would stop the interview for ethical reasons [22]. To ensure confidentiality of the data, all participants were given pseudonyms and code numbers. The interviews were translated by the investigators (linguistic level of communicative knowledge) and then the investigators met with the English teacher and together made linguistic corrections so as not to change the quality and meaning of the statements.

### 2.4. Data Analysis

The audio recordings of the interviews were transcribed verbatim by the nurses who collected the interviews. The transcriptions of the interviews were read and checked against the audio recordings for any discrepancies. Then, according to the chronology of dates, a number was assigned to the respondent. The assigned numbers were used during the qualitative analysis. Respondents were given an abbreviation, where “P” represented the patient and “F” represented family. The respondents’ verbatim transcriptions were read several times. The next step was to categorize the notes by assigning themes. Material from each interview was included in the procedure. Thematic analysis, which is a useful method for exploring the perspectives of different research participants [26], was used. Six themes were identified from the analysis of patient data: “education”, “feelings before the delirium”, “pain”, “thirst”, “the day after”, and “talking to the family”. Four themes were identified from the data collected among family members: ‘education’, ‘talking to the patient’, ‘return home’, and ‘pain’. The reporting of the survey results follows the COREQ (Consolidated criteria for Reporting Qualitative research).

## 3. Results

Finally, eight out of 11 pairs (patient-caregiver) were selected for the study, which represents 72.7%. Eight interviews were conducted with past ICU patients who developed delirium during hospitalization and their family members. The mean age of the participants was 71 years (range 61–80). Of the 8 patients, 2 (25%) were female and 6 (75%) were male. The relationships of the 8 caregivers to the patients were wife (in 4 cases), daughter (in 2 cases) and son (in 2 cases). The average length of patient stay in the ICU was 24 (range 16–33). All patients were diagnosed with delirium by the nurse and after consultation with the doctor based on the NuDESC PL. During the analysis, we distinguished six themes (Table 2) which were discussed and presented in narrative form, citing excerpts from participants’ statements.

### 3.1. Education

This category refers to relatives’ and patients’ experiences of knowledge and information about delirium. For patients and their relatives, the term delirium was unfamiliar. When describing their experiences, most patients reported a lack of information about the possibility of it occurring after surgery.

“*I had no idea that such things could happen to me.*”

(P2)

“*Nobody had told me that various things can happen, so how was I supposed to know?*”

(P4)

Moreover, patients and family members were not at all aware of what the delirium was about or what was happening, but expressed a need for information from medical staff about the possibility of a change in the patient’s behavior:

“*Nobody had told us before that we should prepare for something like that.*”

(F1)

“*Nobody told me that I might be seeing strange things, etc. I think before such a procedure someone should tell us about things which may have an impact on us after the surgery.*”

(P6)

“*I was mad at my husband, but on the other hand I am a little disappointed with the people who should have warned me against such situations.*”

(F7)

Explaining what delirium is and the changes that can occur in a patient’s behavior, information that it is a condition that can occur in a patient hospitalized in the ICU can reduce anxiety among both patients and family. Patients stated that if they had been told about the possibility of delirium, it could have had a positive impact on their wellbeing:

“*Before the procedure nobody told me that this can happen after anesthesia. Maybe if I had known, I would handle it better*.”

(P1)

“*If I had known that something like this could happen to me, maybe I would have been less anxious. Or I would have asked somebody for some sedatives.*”

(P5)

Patients and their families expressed the need for knowledge about delirium. Education and support would help to prepare the family for an encounter with a patient who is experiencing delirium. One family member stated that lack of knowledge and awareness of the possibility of delirium made him nervous:

“*It’s a pity that nobody prepared us for this kind of situation, then I wouldn’t be so upset.*”

(F5)

### 3.2. Feelings before Delirium

This category refers to patients’ experiences related to the period before the delirium symptoms. During the interview, participants were asked about the feelings they had before the delirium episode. As described by most patients, these were mainly negative experiences. For some, delirium was manifested by verbal aggression towards medical staff. Others described the condition as one that could be interpreted as another reality.

“*I remember very well that I caught the nurses’ hand when she was giving me the medicine through this big cannula. Then I remember cursing.*”

(P3)

“*I do not even want to remember it all. I felt like in a movie.*”

(P6)

On the other hand, two patients experienced hallucinations that caused fear for their lives and mental deterioration:

“*I remember I started yelling and the nurse came to me quickly. I was seeing various weird things. I thought someone was trying to poison me instead of helping me.*”

(P1)

“*Then I remember my anger intensified. Later I remember those horrible faces running around the room.*”

(P8)

Several patients admitted that they did not remember delirium events accurately, but only single facts:

“*I don’t remember anything that I did, but I do remember that I was angry at everyone because I couldn’t sleep.*”

(P2)

“*Nobody would give me water, and I know I wanted to get out of the bed. I do not remember what happened after.*”

(P7)

### 3.3. Pain and Thirst

Another topic addressed during the interviews was pain and thirst. In this category, the questions to the patients were focused on the experience of pain and feeling thirsty. It appears that some patients experienced pain complaints that did not subside even after pharmacotherapy. A patient expressed that despite pain medication, the pain was consistently severe.

“*That day everything hurt me so much. I kept receiving something for the pain, but it would not go away.*”

(P5)

Additionally, the wife of one of the patients referred to strong complaints of pain, which made the patient anxious, agitated, and exacerbated delirium symptoms.

“*My husband complained about the pain over the phone. When I came to visit, his pain was a little less intense, but be started to be anxious. What happened later was totally devastating for me.*”

(F7)

Another experience described by patients was a strong feeling of thirst that was unbearable. The thirst caused them to become more aggressive and anxious.

“*I remember I was so awfully thirsty that I did not care if I suffocated or vomited. And nobody wanted to give me water, and I know I wanted to get out of the bed.*”

(P7)

“*I was thirsty all the time. I started to be upset because of that. But the woman gave me an ice cube and I calmed down a little. Everything would be good, but when after a few hours I asked for another ice cube, a different nurse yelled at me that I could not have them so often.*”

(P8)

In some patients, thirst was so severe that sedatives had to be used. Excessive thirst was also noted by the patients’ carer.

“*All my dad was talking about was drinking water. I even began to wonder whether he is right in the head. When I called in the evening, the doctor said that they had given him sedatives because they could not handle him.*”

(F8)

### 3.4. Feelings after Delirium

Another category refers to the feelings experienced by patients after an episode of delirium. Delirium is classified into three subtypes depending on its course (hypoactive, hyperactive, or mixed form). The feelings that accompanied the patient after an episode of delirium were described as shame, embarrassment. Especially then, patients need a lot of support from medical staff.

“*I was very embarrassed on the next day. I felt as if everyone was watching me and that everybody remembered what I had done the day before.*”

(P1)

“*On the next day, the doctor told me what had happened and still I cannot believe it. Nurses are giving me lots of support. I even cried in front of one of them out of shame and she consoled me.*”

(P2)

“*On the next day I wanted to disappear.*”

(P6)

“*I apologized to everyone profusely afterwards. Everyone was saying it is all right, but I felt like a criminal.*”

(P7)

Some patients do not remember the events that took place during the delirium episode. On the other hand, they were drowsy the day after the episode.

“*On the next day I slept till late and I don’t remember much.*”

(P3)

“*If on the next day the Professor had not told me what had happened, I would not know. Oddly enough, I was very sleepy already in the morning, and this made me wonder. Doctors told me that this happens sometimes.*”

(P4)

“*The only thing I remember from the next day is that I woke up around dinner time and I did not feel any pain, but I knew everyone was looking at me funny because of what I had done the day before. A psychologist came to me. I talked to her about it all.*”

(P5)

Symptoms such as sleepiness following an episode of delirium were also noted by the patients’ carers and were a cause for concern for the family.

“*When I called my husband in the morning, they told me he was sleeping. I was surprised because he never slept so much. At 10 he was usually at work or doing some DYI in the garage. I called the doctor right away and he told me that something had happened with my husband. When I visited him, he asked me to buy flowers and give them to the nurses, because he had caused some trouble last light.*”

(F4)

### 3.5. Talking to the Family/Patient

This category related to patients’ and their families’ conversations about the events that took place during the delirium episode. Patients felt shame related to the events that took place during the delirium. This was a difficult experience for them. Patients who talked to their relatives about delirium-related events asked for discretion. They could count on the support of family members and medical staff.

“*I told my son what had happened. He knew anyway from the doctor because I could not talk over the phone. I asked him not to tell my wife. My son supports me. He knows me and knows it was not on purpose.*”

(P1)

“*I told my wife and she did not believe me. But I know she supports me anyway.*”

(P4)

“*I told my daughter about everything, but she was very upset, so I asked her to be discrete. My daughter gives me lots of supports. The nurses did not make me feel like I did something wrong, although I know that was the case.*”

(P6)

“*I told my son about it. It is good to have someone to talk to.*”

(P8)

However, there are patients in whom the sense of shame was so great that they did not want to talk about the delirium incident with relatives. Family members also noticed this. Relatives respected this fact and did not want to bring up the subject of delirium, so as not to cause distress to the relative.

“*I never told my family about it, simply because I am ashamed.*”

(P2)

“*Mom did not tell us right away that something wrong had happened. When I went to visit her, she seemed strange, but I thought this was because of the medications. Now mom does not mention the subject, so I do not want to irritate her by recalling it.*”

(F2)

Patients’ families often first hear about an episode of delirium from ICU doctors.

“*The doctor talked to my family afterwards and told them what had happened. My wife could not believe it, and my son was having a laugh. Now I talked about this with the doctor and he told me that such things happen. Fortunately, I did not hurt anyone. I do not know what came into me.*”

(P3)

“*My family knows what happened because the doctor talked to them during their visit.*”

(P5)

A change in the behavior of ICU patients often comes as a major surprise to the family and can affect their wellbeing. Some described that the patient had become a different person. Changes in their relative’s behavior, aggression, agitation transferred to increased fear and anxiety in the patient’s relatives, and in some relatives to feelings of nervousness towards their relative.

“*When my husband told me that he had almost beaten up the nurses, I thought he went mad. My son and me could not believe it. He is such a calm person. I never had such problems with him.*”

(F3)

“*My mom told me that she had been seeing things, screaming,* etc. *She was very worried about it all, so my sister and me are giving her as much support as we can.*”

(F6)

“*The doctor told me that he had been yelling, spitting, and cursing. I was furious at him.*”

(F7)

### 3.6. Return Home

Returning patients home after a stay in an ICU unit can be stressful for both patients and their carers. However, patients’ families felt that returning home would have a positive impact on their relatives and that the family environment would make it easier for them to forget what happened during hospitalization. Some explained the change in the patient’s behavior as a natural thing after anesthesia.

“*I am not concerned about my mom’s behavior at home because I think it was all because of the anesthesia and that now everything is back to normal.*”

(F2)

“*I know when he returns home, he will be more at ease. We never again talked about what had happened here.*”

(F3)

“*I know that when I take my father home, he will start forgetting that this ever happened and it will be easier for him.*”

(F8)

## 4. Discussion

An attempt was made to show the experiences of patients and family members regarding an episode of delirium. Patients who had been hospitalized in the intensive care unit, together with their family members, talked about their experiences of experiencing an episode of delirium.

Our study, consisting of a semi-structured interview, showed that the main problem for both patients and their families was the lack of education about delirium and the need for information in this area. Most patients and their families repeated that they had not been informed about the possibility of a delirium episode. In a study by Huang et al., the experience of carers of ICU patients with delirium was described as ‘Sailing in a sea of perplexity’. This was due to unfamiliarity with the ICU environment and a lack of knowledge about the care of patients with delirium [21]. In our study, many patients pointed out that if they had been informed earlier about the possibility of changing their behavior, they may have found it easier to manage it. This points to the need for improved communication between medical staff and patients. Similarly, in a study by Huang LJ et al., difficulties in decision-making among caregivers were due to limited knowledge of the patient’s medical needs and limited communication with ICU staff [21]. In a study by Bohart et al., relatives described the need for both written and verbal information about delirium from medical staff [20]. This highlights the need to implement information and education programs in ICUs about delirium. However, it should not be forgotten that programs should also include a form of face-to-face dialogue with medical staff, as demonstrated in a study by Toye et al. This study also showed that relatives sought information about delirium on their own, as medical staff did not meet their needs [27].

Before the onset of delirium, patients experienced many negative emotions. Patients experienced increased anger, hallucinations. This indicates the need to systematically monitor and assess patients for delirium, to detect clinical symptoms of delirium as soon as possible, and implement appropriate interventions. These observations are consistent with the experience of patients in the study by Pandhal JK et al. [28].

People who experienced delirium while in the intensive care unit often felt ashamed and embarrassed by their behavior. Similarly, families of delirium patients were concerned about the altered behavior of their relatives. Family members noticed strange patient behaviors during visits. They described these as restlessness and confusion, which they explained by the anesthetic used the day before. In a study by Day et al., families described that their relative became strange and unfamiliar [29]. Patients’ family members emphasized that they were not prepared for the disturbance of consciousness after the procedures. According to studies by Grover S et al. and Morandi et al., changes in patients’ mood can lead to shock and emotional distress felt by family members [30,31]. In addition, clinical symptoms of delirium can cause distress and frustration among caregivers of ICU patients [32,33]. Findings indicate that patients and their families experience distress after delirium and that family members show a predisposition to depressive symptoms [34,35].

Another aspect noted by patients is that most of them do not remember what happened after the onset of delirium. Such experiences may be related to the pharmacotherapy used to alleviate the delirium. Patients’ statements that they felt drowsy and weak the next day were consistent with their families’ observations. Similar experiences were reported by patients and their families in a study by Schmitt et al. In this study, according to the authors, this condition may also be related to the pharmacotherapy used [4].

It should also be noted that most patients felt uncomfortable and had concerns about confessing to their family that they had experienced an episode of delirium. This shows that, despite the high prevalence of delirium, it is still an under-reported topic in intensive care units. Psychological support should therefore be provided to the patient to rework the events related to the delirium episode.

Additionally noteworthy is the statement of patients who emphasized that the thirst accompanying their stay in the ICU was so difficult to bear that they became anxious and aggressive. Patients’ families also highlighted that their relatives mentioned feeling increased thirst. In a study by Keijzer et al., 11% of patients reported increased thirst and obsessions with drinking [36]. The development of aggression and anxiety due to increased thirst is potentially modifiable and can be avoided with simple interventions, or example, giving the patient an ice cube to suck on or moistening the mouth.

An interesting finding of our study is that most families had no concerns about their relative’s return to the home environment. Additionally, they claimed that returning home would make it easier for their relatives to forget the delirium-related events during their hospitalization in the ICU.

## 5. Limitations

This study has several limitations. Firstly, the participants who were included in the study were limited to those who consented to participate in the study and self-reported to us 1 month after hospital discharge. Therefore, the perspective captured in this study may be narrowed. In addition, due to experiencing delirium, patients may not accurately remember events that occurred during hospitalization. Secondly, the study was conducted in only one cardiac ICU. Thirdly, a small number of participants were included in the study. This is partly because a stay in the ICU is a traumatic experience and many patients do not want to revisit their memories of the time of hospitalization. Conducting additional interviews could potentially reveal added information, perspectives, and experiences of delirium.

## 6. Conclusions

Understanding the perspective and experiences of patients and their families related to an episode of delirium is an important part of the management of delirium. Education and information about delirium in the ICU for patients and their families is inadequate. Medical staff should inform patients and their families about the possibility of delirium episodes. ICU patients and their families show a need and desire to be informed and educated about delirium. Most patients feel embarrassed and ashamed about events during a delirium episode. Patients fear the reaction of their families when delirium occurs. Patients’ families are not concerned about their loved ones returning home and believe that the home environment will allow them to forget the delirium events more quickly during hospitalization. The study found that the feelings of patients and their families provide an important perspective and influence their wellbeing.

## 7. Implications for Practice

Delirium affects not only critically ill patients but also relatives of patients, which can increase anxiety and stress for carers. Increasing medical staff awareness of delirium and its negative impact on ICU patients and their carers is an important part of managing delirium in critically ill patients. With the heavy workload associated with the nature of the ICU, delirium becomes secondary. Educating medical staff and demonstrating the benefits of delirium prevention could help to increase awareness, the importance of regular delirium assessment. Delirium should not be a topic ignored by medical staff when talking to patients and their carers. Patients should be informed of the potential for behavioral changes that are associated with delirium, while providing an atmosphere of support and understanding. Education about delirium should also be directed to the family caring for the ICU patient. Leaflets, posters specially adapted for families, can be used for this purpose. However, it should not be forgotten that indirect education, based only on ready-made tools, should not replace the personal contact of the medical staff with the patient and their family, but only complement it. Many patients feel ashamed and embarrassed about their behavior during delirium. They are often unwilling or not ready to talk about it with their relatives. It would be useful to consider providing these patients with support, e.g., by talking to a psychologist. Severe thirst increases aggression and anxiety in ICU patients. Nursing staff should implement interventions to reduce thirst, e.g., by giving ice cubes to suck on, moistening the patient’s mouth.

## Figures and Tables

**Table 1:** Questions for patients and families.

**Questions for the patient**
Do you remember how you behaved and what you did during the hard time in the ICU? What did you feel the next day after you experienced an emotional disorder? Did someone talk to you before the surgery about the possibility of an emotional disorder? Did you talk to your family about your behavior? Did you get support after the emotional disorder?
**Questions for the family**
Did your relative tell you how he/she behaved during the difficult experiences in the ICU? What did you feel when you found out about the emotional disorder experienced by your relative? Did someone talk to you before the surgery about the possibility of an emotional disorder? Did you talk to your relative about your behavior? Did you get support after your relative’s emotional disorder? Are you concerned about taking your relative home knowing that he/she experienced an emotional disorder while being in the ICU?

**Table 2:** Themes identified during the analysis.

Themes
1. Education
2. Feelings before the delirium
3. Pain and Thirst
4. The day after
5. Talking to the family/patient
6. Return home

## Data Availability

The authors declare that the data of this research are available from the correspondence author on request.
